# Influence of grand-mother diet on offspring performances through the male line in Muscovy duck

**DOI:** 10.1186/s12863-015-0303-z

**Published:** 2015-12-21

**Authors:** Jean-Michel Brun, Marie-Dominique Bernadet, Alexis Cornuez, Sophie Leroux, Loys Bodin, Benjamin Basso, Stéphane Davail, Mathilde Jaglin, Michel Lessire, Xavier Martin, Nadine Sellier, Mireille Morisson, Frédérique Pitel

**Affiliations:** UMR INRA, Génétique, Physiologie et Systèmes d’Elevage, INRA, 31328 Castanet Tolosan, France; INPT ENSAT, Génétique, Physiologie et Systèmes d’Elevage, INRA, 31328 Castanet Tolosan, France; INPT ENVT, Génétique, Physiologie et Systèmes d’Elevage, INRA, 31328 Castanet Tolosan, France; Institut National de la Recherche Agronomique, Unité Expérimentale des Palmipèdes à Foie Gras, UE89, 40280 Benquet, France; UMR5254 IUT des Pays de l’Adour-CNRS, 40004 Mont de Marsan Cedex, France; Institut National de la Recherche Agronomique, UR83 Unité de Recherche Avicole, 37380 Nouzilly, France; Institut National de la Recherche Agronomique, Pôle d’Expérimentation Avicole de Tours, UE1295, 37380 Nouzilly, France; Present addresses: ITSAP-Institut de l’Abeille, Site Agroparc, 84914 Avignon, France; UMT Protection des Abeilles dans l’Environnement, CS 40506, 84914 Avignon, France

**Keywords:** Environment, Diet, Methionine, Duck, Multigenerational, Epigenetics

## Abstract

**Background:**

In mammals, multigenerational environmental effects have been documented by either epidemiological studies in human or animal experiments in rodents. Whether such phenomena also occur in birds for more than one generation is still an open question. The objective of this study was to investigate if a methionine deficiency experienced by a mother (G0) could affect her grand-offspring phenotypes (G2 hybrid mule ducks and G2 purebred Muscovy ducks), through their Muscovy sons (G1). Muscovy drakes are used for the production of mule ducks, which are sterile offspring of female common duck (*Anas platyrhynchos*) and Muscovy drakes (*Cairina moschata*). In France, mule ducks are bred mainly for the production of “foie gras”, which stems from hepatic steatosis under two weeks of force-feeding (FF). Two groups of female Muscovy ducks received either a methionine deficient diet or a control diet. Their sons were mated to Muscovy or to common duck females to produce Muscovy or Mule ducks, respectively. Several traits were measured in the G2 progenies, concerning growth, feed efficiency during FF, body composition after FF, and quality of foie gras and magret.

**Results:**

In the G2 mule duck progeny, grand-maternal methionine deficiency (GMMD) decreased 4, 8, and 12 week body weights but increased weight gain and feed efficiency during FF, and abdominal fat weight. The plasmatic glucose and triglyceride contents at the end of FF were higher in the methionine deficient group. In the G2 purebred Muscovy progeny, GMMD tended to decrease 4 week body weight in both sexes, and decreased weight gain between the ages of 4 and 12 weeks, 12 week body weight, and body weight at the end of FF in male offspring only. GMMD tended to increase liver weight and increased the carcass proportion of liver in both sexes.

**Conclusion:**

Altogether, these results show that the mother’s diet is able to affect traits linked to growth and to lipid metabolism in the offspring of her sons, in Muscovy ducks. Whether this transmission through the father of information induced in the grand-mother by the environment is epigenetic remains to be demonstrated.

**Electronic supplementary material:**

The online version of this article (doi:10.1186/s12863-015-0303-z) contains supplementary material, which is available to authorized users.

## Background

In animal breeding, maternal and even grand-maternal effects, in addition to the direct effects of the genes transmitted to the offspring, have been postulated, modelled and estimated for a long time [1, 2]. The mother contributes to the offspring phenotype through the genes she passed on to the next generation, but also through maternal effects, independent from the genes the offspring has received from his mother. The renewed interest epigenetics has acquired in biological sciences in the past few decades has in many cases given a biological basis to non-genetic multigenerational effects [3, 4]. However, while multigenerational effects of maternal environment are well recognized, whether epigenetic or not, those of paternal environment are more rarely considered, even though they more certainly represent epigenetic phenomena, or “transgenerational inheritance”. Over one generation, several animal and epidemiological studies on various nutrition and life-style related conditions have reported an effect of the paternal environment on the offspring phenotype (pre-mating fasting of male mice [5]; high fat diet in male rats [6]; see [7, 8] for a review). Non-genetic effects involving the male germ-line have also been documented by either epidemiological studies in human such as the prenatal exposure of fathers to the Dutch famine [9], exposure of paternal grand-fathers [10, 11] or grand-mothers [12] to different food supplies in Sweden, or animal experiments such as the effects of endocrine disruptors on male fertility in the rat [13]. Whether these phenomena also occur in birds has not been investigated yet [14].

The objective of this study was thus to investigate non-genetic multigenerational effects induced by the environment in duck, the environment effector being here a dietary methyl-donor deficiency. In France, mule ducks, which are hybrids of common female ducks (*Anas platyrhynchos*) and Muscovy drakes (*Cairina moschata*) are bred for the production of “foie gras” and of “magrets”, the pectoral muscle (with skin) of force fed ducks. Force-feeding leads to a hepatic steatosis, with a tenfold increase in liver weight within two weeks, from 12 to 14 weeks of age. The idea of investigating multigenerational effects in the duck stemmed from a parallel between steatosis in duck and obesity in human and in mice, which is often associated to multigenerational effects and to epigenetic mechanisms ([7, 15], see [16] for a review). A methyl-donor limitation was applied to the Muscovy dams (G0) before and during the conception of their sons (G1), through a methionine restriction of the diet. The multigenerational effects were evaluated on G2, either purebred Muscovy ducks or hybrid mule ducks issued from these G1 sires.

## Methods

All procedures were conducted in accordance with guidelines for Care and Use of Animals in Agricultural Research and Teaching (French Agricultural Agency and Scientific Research Agency; approval number of the experimental farm: B40-037-1; Ethics committee approval n°00066.03 from the C2EA-73 “Comité d’Ethique Aquitaine Poissons Oiseaux”).

### Animals and experimental design

**Fig. 1:**
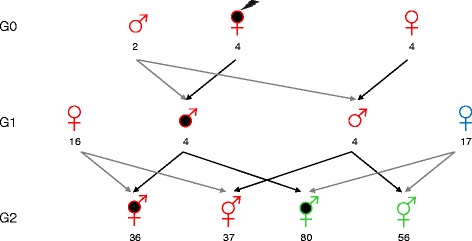
Experimental design. In G0, four females were fed a Met-deficient diet while four other females were fed a control diet. They were inseminated with semen of two Muscovy drakes in order to produce G1 drakes. A total of 25 G1 ducks of both sexes were raised, of which eight males were kept for reproduction, four from each diet group of the G0 dams. The procreation of the G2 mule ducks was performed by artificial insemination with the semen of G1 Muscovy drakes to common duck females. The procreation of G2 purebred Muscovy ducks was performed by artificially inseminating the semen of G1 Muscovy drakes to Muscovy females. Black: with Met-deficient diet, hatched: having an ancestor with Met-deficient diet, red: Muscovy duck, blue: Common duck, green: Mule duck. The number of individuals in each group is indicated

The experiment was conducted at the INRA UEPFG experimental farm for waterfowl (Benquet, France) between 2010 and 2013, and involved three generations of animals, G0, G1, and G2 (Fig. 1).

In G0, 8 Muscovy female ducks of the R71M strain were purchased (Grimaud Frères S.A.), raised at UEPFG and allocated to two diet groups when placed in individual reproduction cages at the age of 10 weeks until the age of 35 weeks. Both groups were fed a growing diet until 17 weeks of age, followed by a reproduction diet until the end of breeding. Females of the first group (*n* = 4) were fed a Met-deficient diet. The other four females were fed a control diet. The nutritional characteristics of the growing and reproduction diets, which were produced at the INRA PEAT experimental mill (Nouzilly, France) are given in Table 1. The growing and the reproduction diets were produced from the same base, but the Met contents was 2.6 g/kg for the Met-deficient diet and 4.2 g/kg for the control diet, the latest being in conformity with the recommendations. The diets were given as pellets.

**Table 1:** Composition of the Met-deficient diets^1^ of the G0 female Muscovy duck

	Growing diet (9–15 weeks)	Reproduction diet (beyond 15 weeks)
Ingredients g/kg		
Maize	501	461.8
Soybean meal	196.3	222.3
Wheat	200	200
Wheat bran	53.3	26.8
Soybean oil	20	10
Phosphate Dicalcium	13.4	16.8
Limestone	7.78	54.8
Trace elements	5	4
NaCl	3	3
Lys Hcl	0.22	0.5
Expected nutritional composition		
Metabolisable Energy, Kcal/kg	2900	2700
Protein, g/kg	160	165
Lys, g/kg	7.8	8.5
Met, g/kg	2.56	2.61
Met + Cyst, g/kg	5.59	5.65
Try, g/kg	1.79	1.86
Thr, g/kg	5.96	6.16
Ca, g/kg	8.55	27
P, g/kg	3.51	4.01
Choline chloride, g/kg	0.55	0.55

^1DL-^Met was added to form the control diet

At the age of 25 to 26 weeks the females were inseminated with semen of Muscovy drakes of the INRA66 strain in order to produce G1 drakes. Each drake inseminated two Met-deficient and two control females. A total of 25 G1 ducks of both sexes were raised, of which eight males were kept for reproduction, four from each diet group of the G0 dams. The G1 males were from three different mothers in each group. The procreation of the G2 mule ducks was performed by artificially inseminating the semen of G1 Muscovy drakes to common duck females of the INRA444 strain. Two batches were produced (2012 and 2013). The procreation of G2 purebred Muscovy ducks was performed by artificially inseminating the semen of G1 Muscovy drakes to Muscovy females of the INRA66 strain. Only one batch was produced (2013). A mating plan was designed and controlled at each generation: the inseminations were performed following a nested mating design: the semen from each male was used for inseminating its own mates.

The G2 mule ducks and Muscovy ducks were fed ad libitum a commercial starting diet from 0 to 4 weeks of age, followed by a commercial growing diet until 12 weeks of age. From the age of 8 weeks until 12 weeks, all the G2 ducks underwent preparation for force-feeding in order to expand their crop by restricting the duration of access to the feeders and making the ducks ingest their ration quickly. They were then force-fed in collective cages of four ducks of the same sex, genetic background and diet groups. Force-feeding (FF) consisted of two meals per day for 13 d (a total of 25 meals) with a mixture of 35 % maize flour, 25 % maize grain, and 40 % water. A specific FF planning was applied to mule ducks (the same for both sexes), to male Muscovy ducks and to female Muscovy ducks, with a planned total feed quantity at 9685 g (dry matter), 7265 g and 5760 g for the three types, respectively. When the previous meal had not been digested by a duck it was either fed a half-meal or skipped the meal. Slaughter occurred at the end of force-feeding, at 14 weeks of age, in the slaughter room of the experimental farm. The ducks were bled after electronarcosis and plucked. The carcasses were refrigerated for 24 h at 4 °C, and then eviscerated. Livers and magrets were removed. The magrets were dissected into muscle and skin (including subcutaneous fat). Samples of livers were taken in order to evaluate the technological yield of liver at sterilization, generally denoted by “melting rate” according to the procedure described by [17].

### Traits studied

The focus was put on the G2 mule and Muscovy ducks. However, several traits which could have been affected by the Met restriction were recorded in the G0 and G1 generations. Concerning G0 Muscovy females, laying was recorded all over the laying period. Fertility and hatchability were recorded during the reproduction periods. Concerning G1, male and female offspring were weighed at the ages of 24, 43, 65 and 95d. The 8 males retained for reproduction were also weighed at 143d of age.

Concerning the G2 mule and Muscovy ducks, body weight at 4, 8, and 12 week of age were measured and the weight gains between consecutive ages were calculated. Individual feed consumption during FF was recorded for each meal and total feed consumption during FF was calculated. Body weight at the end of FF was recorded and weight gain during FF was calculated, along with the ratio (ingested feed/ weight gain) indicating feed efficiency during FF. Carcass was weighed and carcass yield was calculated. Liver weight, the carcass percentage of liver and the melting rate were recorded. Abdominal fat weight and its carcass percentage were evaluated. Magret weight, carcass percentage of magret, and the percentage of ‘skin + subcutaneous fat’ in the magret, an indicator of overall subcutaneous fatness of the duck, were also recorded. The concentration of two plasma parameters, glucose and triglycerides, was determined from blood samples taken three hours after the meal, at two stages of the FF period: in the middle (after the 12th meal) and at the end (after the 24th meal).

### Statistical analyses

Laying and reproductive output of G0 females were analyzed by analysis of variance (ANOVA) according to the diet. The weights of G1 offspring during growth were analyzed by ANOVA according to the maternal diet, the sex, and an interaction between both factors. Concerning G2, the mule duck traits and the Muscovy duck traits were analyzed separately, because of i) the different time span of both genetic types (two years in mule ducks vs. one year in Muscovy ducks), ii) the specific high sexual dimorphism of body weight in Muscovy ducks and iii) hence, the potentially specific interactions involving year and sex. The mule duck traits were analyzed by ANOVA with the fixed effects of grand-maternal diet, year, sex, and paternal grand-sire (2 levels). In a first step, all 2 by 2 interactions were introduced in the model except those involving the paternal grand-sire. The interactions ‘diet by year’ and ‘diet by sex’ were not significant. The model retained was thus a model without interactions for all traits except for liver weight, carcass percentage of liver and liver technological yield, for which a ‘year by sex’ interaction was included in the model.

The model retained for the analysis of G2 Muscovy ducks included the fixed effect of grand-maternal diet, sex, the paternal grand-sire, and an interaction between grand-maternal diet and sex. In the analyses of the G2 progeny traits, we tried to partition at best what is due to genetic inheritance vs. diet-related parental effects. This could have been achieved by taking into account a dam effect or a full-sib family effect. However, since the design involved dams nested within sires which were themselves nested within the diets,, this would have neither changed the estimate of the diet effect, nor prevented an eventual confounding. Actually, this confounding can be partially removed only through effects which are crossed and not nested with the diet effect. The only genetic effect that was crossed with the diet was the paternal grand-sire of the G2 individuals and it was included in the model.

The ANOVAs were performed using the PROC GLM procedure of SAS (SAS Inst., Cary, NC, USA).

## Results

### Zootechnical results at G0 and G1 generations

Met deficiency showed no significant effects on laying rate, fertility or hatchability.

Maternal diet had no significant effect on the weights of G1 progeny at any age and there was no significant diet by sex interaction. The eight males (four Met-deficient and four Control) which were utilized as breeders to procreate the G2 offspring showed no significant weight differences between diets. Their 143 d-body weight was, on average, 4244 ± 109 g for the Met deficient diet and 4182 ± 109 g for the control diet.

### Effects of grand-maternal Met-deficiency (GMMD) on G2 mule ducks

Table 2 gives descriptive statistics of the G2 mule duck traits, the statistical significance of the factors of the ANOVA and the least square means of the G2 mule duck traits, according to grand-maternal diet, for traits showing significant diet effects in this progeny type. For comparison purposes between both progeny types, means are also given for the traits showing a significant diet effect in the Muscovy progeny. In the following text, G2 ducks with a Met-deficient grand-mother will be referred to as GMMD ducks.

**Table 2:** Results obtained on G2 mule duck traits. Statistical significance of the effects of grand-maternal (GM) diet, year, sex and year by sex interaction on G2 mule duck traits (paternal grand-sire effect is not given), and least square means (± SE) of GM diet^a^

	N	GM diet	Year	Sex	Year x sex	Met-deficient	Control
Growth traits							
Body weight, 4 weeks	133	***	ns	***	-	1326 ± 10^d^	1397 ± 11^c^
Body weight, 8 weeks	134	***	***	***	-	2814 ± 19^d^	2913 ± 21^c^
Body weight, 12 weeks	133	*	***	Ns	-	3390 ± 23^d^	3458 ± 25^c^
Weight gain, 4–8weeks	133	ns	***	ns	-		
Weight gain, 8–12weeks	133	ns	ns	***	-	577 ± 19	545 ± 21
Weight gain, 4–12weeks	132	ns	***	***	-		
Force-feeding (FF) traits							
Weight gain during FF	130	**	ns	***	-	1609 ± 23^c^	1502 ± 25^d^
Body weight, 14 weeks (end FF)	131	ns	**	***	-	5016 ± 30	4960 ± 33
Feed consumption during FF	131	ns	***	***	-	14071 ± 48	14026 ± 53
Ratio FC/WG during FF	129	***	ns	***	-	8.78 ± 0.15^c^	9.58 ± 0.17^d^
Carcass traits							
Carcass weight (CW)	129	ns	*	***	-		
Magret^b^ weight	131	ns	ns	*	-	367.1 ± 3.4	364.5 ± 3.7
Ratio (magret/carcass)	128	ns	*	ns	-		
Magret muscle weight	131	ns	ns	ns	-		
Ratio (fat + skin/magret)	131	ns	ns	ns	-		
Abdominal fat weight (AF)	131	*	ns	**	-	156.7 ± 2.7^c^	146.5 ± 2.9^d^
Ratio AF/CW %	128	*	ns	ns	-	3.45 ± 0.05^c^	3.27 ± 0.06^d^
Liver weight (LW)	131	ns	ns	ns	***		
Ratio (LW/CW)	128	ns	ns	**	**	12.1 ± 0.3	11.9 ± 0.3
Liver melting rate	131	ns	***	***	**		
Blood metabolites							
Glucose mid-FF (g/L)	129	ns	ns	**	-		
Glucose end-FF (g/L)	105	+	ns	***	-	3.27 ± 0.19	2.83 ± 0.20
Triglycerides mid-FF (g/L)	129	ns	***	ns	-		
Triglycerides end-FF (g/L)	105	*	ns	ns	-	0.85 ± 0.06^c^	0.64 ± 0.07^d^

^a^Least square means of GM diets are given for traits showing an effect of GM diet (*P* < 0.10) and also for traits showing a significant GM effect in the Muscovy duck progeny (*P* < 0.05)

^b^“Magret” is the *Pectoralis major* with skin of a force-fed duck; FF = Force Feeding

***P < 0.001; **P < 0.01; *P < 0.05; +*P* < 0.10; ns: not significant; ‘-‘interaction not included in the model

^c,d^Values within a row with different superscripts differ significantly at *P* < 0.05

**Fig. 2:**
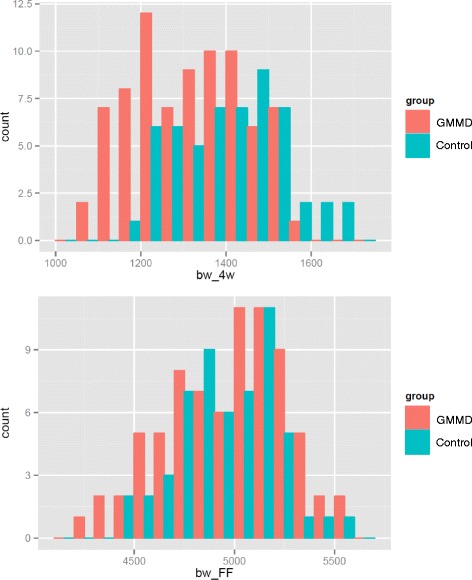
Body weight differences between control and GMMD groups at two ages, in mule ducks. In G2 mule ducks, the depressive effect of grand-maternal Met restriction on 4weeks-body weight is offset afterward by a positive effect on weight gain until the end of force-feeding. Body weight (g) distribution is shown for GMMD (red) and control (blue) groups. Up : body weight at 4 weeks of age. Down : body weight at 14 weeks of age, at the end of force feeding

GMMD had a significant effect (*P < 0.05*) on 4, 8, and 12 week body weights of mule ducks, decreasing body weight by 5 %, 3.4 %, and 2 % at these three ages, respectively. This effect was no longer observed after FF, at the age of 14 weeks (it even showed the reverse trend) because weight gain during FF was significantly higher for GMMD mule ducks (*P < 0.01*, Fig. 2).

Feed consumption during FF was not different between the two diet groups, indicating that they had the same ability to ingest the standardized FF doses. Consequently, feed efficiency during FF was higher in the GMMD group (*P < 0.001*).

Abdominal fat deposits were higher in the GMMD group (*P < 0.05*), as absolute value and as percentage of the carcass.

The plasmatic triglyceride content showed a significant effect of GMMD at the end of FF, but no effect at mid-FF. It was significantly higher in the GMMD group *(P* < 0.05). The glucose content at the end of FF showed a trend toward a higher value in the deficient diet group (3.27 ± 0.19 g/L for the GMMD group vs. 2.83 ± 0.20 g/L for the Control group, *P* < 0.10).

It is noteworthy that GMMD had no significant effects on several carcass traits: carcass yield, magret weight, and carcass percentage of magret were not altered. Subcutaneous fatness of the whole body, as indicated by the subcutaneous fatness of the magret was not either altered. At last, liver weight, carcass proportion of liver, and technological yield of the liver were not altered.

As tested in a preliminary step, there was no significant interaction between the diet and any other factor of the model, and in particular the sex of the mule duck, indicating that the observed effects were not sex-specific (Additional file 1).

Overall, GMMD effects were significant for several traits, repeated in both years of tests, and were not sex-specific.

### Effect of grand-maternal Met-deficiency (GMMD) in G2 purebred Muscovy ducks

Table 3 gives the statistical significance for the factors of the ANOVA and the least square means of G2 Muscovy duck traits, according to grand-maternal diet (and to grand-maternal diet by sex combination in case of interaction between both factors), for traits showing significant effects in this progeny type (and also for that showing significant effects in the mule progeny, for comparison purpose).

**Table 3:** Results obtained on G2 Muscovy offspring. Statistical significance of the effects of grand-maternal (GM) diet, sex and GM diet by sex interaction on traits of G2 Muscovy offspring, and least square means (± SE) of GM diet^a^, given by sex in case of interaction

	N	GM diet	Sex	Diet x sex	Met-deficient	Control
Growth traits						
Body weight, 4 weeks	72	+	***	ns	1421 ± 20	1468 ± 20
Body weight, 8 weeks	70	ns	***	ns	3530 ± 36	3560 ± 34
Body weight, 12 weeks	72	+	***	*	4112 ± 43	4221 ± 41
“: males	37				5269 ± 60^b^	5501 ± 59^a^
“: females	35				2955 ± 61	2941 ± 60
Weight gain, 4-8weeks	70	Ns	***	*		
“: males	36				2678 ± 37^a^	2596 ± 36^b^
“: females	34				1525 ± 38	1589 ± 37
Weight gain, 8-12weeks	70	*	***	***	574 ± 34^b^	660 ± 32^a^
“: males	36				921 ± 50^b^	1202 ± 49^a^
“: females	34				227 ± 51^a^	119 ± 50b
Weight gain, 4-12weeks	72	Ns	****	*		
“: males	37				3609 ± 55^b^	3798 ± 54^a^
“: females	35				1772 ± 57	1708 ± 55
Force-feeding (FF) traits						
Weight gain during FF	70	ns	***	ns	1132 ± 30	1153 ± 30
Body weight, 14 weeks (end FF)	70	*	***	ns	5244 ± 51^b^	5390 ± 51^a^
Carcass traits						
Carcass weight (CW)	71	*	***	ns		
Magret^b^ weight	71	ns	***	ns	437 ± 5^b^	452 ± 5^a^
Ratio (magret/carcass)	71	ns	*	ns		
Magret muscle weight	71	ns	***	ns		
Ratio (fat + skin/magret)	71	ns	ns	ns		
Abdominal fat weight (AF)	71	ns	***	ns	154 ± 5	154 ± 5
Ratio AF/CW %	71		**	ns	3.29 ± 0.10	3.18 ± 0.10
Liver weight (LW)	71	+	***	ns	479 ± 12	451 ± 12
Ratio (LW/CW)	71	*	***	ns	10.3 ± 0.2^a^	9.6 ± 0.2^b^
Blood metabolites	71					
Glucose mid-FF (g/L)	71	ns	ns	ns		
Glucose end-FF (g/L)	71	ns	*	ns		
Triglycerides mid-FF (g/L)	71	**	***	**	0.41 ± 0.02^a^	0.33 ± 0.02^b^
“: males	37				0.32 ± 0.02	0.31 ± 0.02
“: females	34				0.50 ± 0.02^a^	0.35 ± 0.02^b^
Triglycerides end-FF (g/L)	70	*	+	ns	0.59 ± 0.06^a^	0.40 ± 0.06^b^

^a^Least square means of GM diets are given for traits showing an effect of GM diet (*P* < 0.10) and also for traits showing a significant GM effect in the mule duck progeny (*P* < 0.05)

^b^“Magret” is the *Pectoralis major* with skin of a force-fed duck; FF = Force Feding

***P < 0.001; **P < 0.01; *P < 0.05; +*P* < 0.10; ns: not significant. ^a,b^ Values within a row with different superscripts differ significantly at *P* < 0.05

**Fig. 3:**
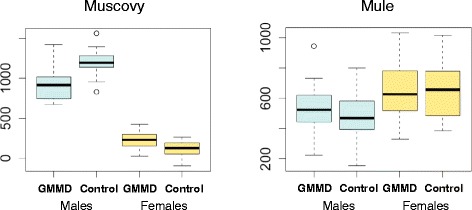
Differences in weight gain (8–12 weeks) between control and GMMD groups according to the sex. Boxplots show the interaction diet x sex in Muscovy ducks (left)

Concerning body weight traits, GMMD tended to decrease 4 and 12 weeks-body weight (*P < 0.10*) and significantly decreased body weight at the end of FF (*P < 0.05*). The effect of GMMD on body weight traits was generally sex-specific, as indicated by trends to interactions between grand-maternal diet and sex, and affected male offspring particularly (Additional file 1). Twelve weeks body weight for example showed a significant interaction (*P < 0.0001*), with no effect on female offspring but a marked effect on male offspring (5269 g and 5501 g for the GMMD and Control ducks, respectively). Four to eight weeks weight gain, and eight to 12 weeks weight gain exhibited significant grand-maternal diet by sex interactions (Fig. 3). From four to eight weeks, GMMD males had a higher weight gain than Control ones (difference at 80 g, *P < 0.0001*), whereas females showed no differences. From eight to 12 weeks, GMMD males had a lower weight gain than Control ones (difference at 280 g, *P < 0.0001*), whereas GMMD females had a higher weight gain than Control ones. Overall, GMMD had a sex-specific effect on weight gain between the ages of 4 and 12 weeks, hampering the growth of male offspring but not that of females. Magret weight was decreased by GMMD, but its proportion in the carcass was not significantly affected. At last, liver weight was higher in GMMD ducks, which had a significantly higher proportion of liver in the carcass.

Whether at mid-FF or at the end of FF, plasmatic glucose content was not affected by grand-maternal diet. By contrast, plasma triglyceride content was influenced by grand-maternal diet at both stages. GMMD increased triglyceride level by 24 % at mid-FF and by 48 % at end-FF. The significant interaction between grand-maternal diet and sex at mid-FF reflected that only the females were affected. The pattern was the same at the end of FF, although not significantly, due to the large standard-deviation of triglyceride content at that stage: no differences between GMMD and Control males (0.46 ± 0.08 g/L and 0.38 ± 0.08 g/L for GMMD and Control males, respectively) but a difference between GMMD and Control females (0.71 ± 0.08 g/L and 0.42 ± 0.08 g/L for GMMD and Control females, respectively). Thus, GMMD had a sex-specific effect on triglyceride content, which was limited to the females.

As for the mule ducks, GMMD effects were significant for several traits in G2 purebred Muscovy ducks, being sex-specific in many cases.

## Discussion

By measuring several production and metabolic traits in purebred Muscovy or hybrid mule ducks of which the paternal grand-mother was fed a methionine-restricted diet, we showed that the mother diet is able to affect the offspring of her sons. The transmission of such effects over two generations through the father was observed in both genetic types for traits such as body weight before force-feeding and triglycerides plasma concentration, while other traits affected by the grand-mother diet were genetic type-specific.

### GMMD effects in mule ducks vs. Muscovy ducks

The effects observed at G2 in mule ducks and in Muscovy ducks share common features: in both genetic types, GMMD decreased body weights during the growth phase from 4 to 12 weeks of age. In the purebred Muscovy ducks, this effect was however limited to the male progeny. A year effect was observed in G2 mule ducks, and particularly a sex by year interaction on liver-related traits. This year effect is likely to be multifactorial since factors influencing these zootechnical traits are numerous, including incubation conditions, temperature and hygrometry during the growth phase, and force-feeding conditions.

Surprisingly, some effects of GMMD were specific of the genetic type of the G2 offspring. The marked effect of GMMD on weight gain during force-feeding and on abdominal fat weight was observed in the mule duck progeny only. The effects of GMMD on magret weight and on carcass proportion of fatty liver were specific of the purebred Muscovy progeny.

The hybrid mule duck progeny and the purebred Muscovy progeny have thus specific responses to GMMD, particularly for growth traits (sex-specific response in Muscovy ducks only) and for FF traits (weight gain during FF in mule ducks only and specific localization of fat deposits).

The sex-specific effects on growth in Muscovy ducks may be linked to the sexual dimorphism of body weight which is particularly marked in this species, as compared to the common duck or to the mule duck [18]. This sexual dimorphism clearly appeared in our study, as for example in the control group: the 12 weeks body weight was 5501 g in males vs. 2941 g in females. The nutritional requirements of males and females are therefore very different, and the fact that GMMD affected particularly weight gain between the ages of 8 and 12 weeks, including the phase of preparation to FF with limited time for feed consumption, could involve sex-specific response group to nutritional restriction. Several studies have shown environmental sex-specific effects, including epigenetics modifications, during embryogenesis (see [19] for a review).

### Grand-maternal Met restriction affected traits associated with lipid metabolism

In contrast with the depressive effect on body weight, GMMD increased traits associated with lipid metabolism such as weight gain during FF and abdominal fat weight in the mule duck progeny, and the carcass proportion of fatty liver in the Muscovy duck progeny. Thus, the diet seems to drive the localization of fat deposits towards abdominal fat in mule ducks, and towards liver in Muscovy ducks. This seems to reinforce the natural differences between these two genetic backgrounds, already observed [20–22]. In birds, the liver is the principal site of lipogenesis, which, in the case of force-feeding, consists of the synthesis of triglycerides from glucose, resulting itself from the digestion of the starch of the feed. In the liver, the unbalance between lipogenesis and lipid exportation leads to liver steatosis. The exported triglycerides are transported as VLDL (Very Low Density Lipoproteins) to peripheral tissues, forming subcutaneous, abdominal, and intra-muscular fat deposits. During FF, most of the body weight gain is made of lipids, not excluding the continuation of muscular growth. Liver weight is a good indicator of liver fat content, since lipids represent 61 % of fatty liver weight [22] and since the correlation between liver weight and liver fat content was found to be 0.95 in a population of mule ducks (X Fernandez, personal communication).

Beyond the lipid synthesis, Met restriction of the grand-mother influenced the body distribution of the triglyceride storage from lipogenesis. Indeed, in the mule duck progeny, abdominal fat weight was altered, but neither was the liver weight nor the subcutaneous fatness of the body after force-feeding. In the Muscovy duck progeny, the body distribution of the triglycerides storage was also altered by GMMD: the ducks from Met-restricted grand-mothers retained more triglycerides in the liver, at the expense of subcutaneous fatness which was decreased, however not significantly. Indeed, the indicator of the overall body subcutaneous fatness, the magret percentage of (skin + fat) was 9.07 ± 0.08 % in the Met-restricted group vs. 9.21 ± 0.08 % in the Control one.

Our data could be viewed in relation with what is observed in mammals where several studies showed that methyl donor deficient diets (MDD diets) affect hepatic metabolism in F0 and/or F1 generations. In rodents for example, Methionine and Choline deficiency favors hepatic steatosis [23, 24], through increased fatty acid uptake and decreased VLDL secretion by the liver [25]. In rats, maternal MDD diet induces hepatic steatosis [26] and changes in liver proteome [27]. In sheep, maternal MDD diet affects the gene methylation level in the liver of F1 offspring [28]. Epigenetic changes have been evoked to explain these effects since methyl donors such as folate, choline, methionine and vitamins B6 and B12, are involved in one-carbon metabolism which releases methyl groups (−CH3) used by methyltransferases to methylate DNA and histone proteins (see [29–31] for reviews). Furthermore, the deficiency of methyl groups may affect the transmethylation of co-regulators of nuclear receptors such as PGC1-α which is a master regulator of lipid metabolism and fatty acid oxidation [26, 29, 30]. More recently, Chen and coworkers identified modifications of DNA methylation in the promoter regions of 1032 genes in liver of F1 offspring from female rats fed with MMD diet [32]. To our knowledge, no such maternal MDD diet studies have been carried out in birds, and our work differs from the ones cited above in the fact that it focuses on the F2 generation and that the non genetic effects of the MMD diet are transmitted through the sons, and thus through their spermatozoa, to the F2 generation. Such effects of female diet transmitted to their grandchildren via their sons have already been reported in humans. Thus studying a cohort of grandchildren of women exposed to 1944–45 Duch famine, Veenendaal and coworkers reported that adult offspring (F2) of prenatally exposed F1 fathers were heavier and more obese than children of fathers who had not been prenatally exposed [9].

### Epigenetic vs genetic effects

It cannot be excluded that part of the differences observed in G2 between the two grand-maternal diet groups, in both the mule duck and the purebred Muscovy progenies, can have a genetic origin. Sampling bias can have occurred in G0, due to the small number of founders, resulting in genetic differences between the two groups of founders. Again, the small number of G1 drakes may add some genetic drift, responsible for an additional error in the G2 progeny mean estimation [33]. Our tests for comparing met-deficient and control G2 offspring did not take the genetic drift into account, but the absence of differences in the average weights of the two groups of G1 males brings some reassurance concerning this putative bias. But the results observed are compatible with the existence of epigenetic effects. Indeed, it is now well documented in mammals that environmental exposures (e.g., toxins, stress or nutritional deprivation) of the G0 generation can influence gene regulations and the adult phenotypes of the G1, G2 (“multigenerational”) and G3 generations (“transgenerational”) through epigenetic mechanisms (e.g., DNA methylation, histone modifications or miRNA) [8, 34–38]. In birds, resources deposited in the egg (e.g., nutrients, hormones, carotenoids, vitamins or RNA transcripts) can also impact newborn fitness and later adult phenotypes of the G1 generation. But the egg composition can also directly affect the G2 generation since the developing G1 generation bears the primordial germ cells that will eventually form a G2 progeny. Hence, the maternal nutritional deficiency may have affected the epigenetic information carried by the G1 drake spermatozoa, as already reported in mammals [39]. This non-genetic inheritance may be partially involved in human metabolic diseases [40]. Epigenetics marks in the Primordial Germ Cells of the developing father may be influenced by the environment of his mother [7]. This transmission of information through the paternal germline may involve modifications of chromatin, small RNAs, or other mechanisms, yet to be deciphered [41, 42].

Contrary to maternal effects [43], paternal effects have received much less attention [44, 45]. Nevertheless, several studies have shown that the father may transmit non-genetic information through spermatozoa epigenetic marks [39, 46, 47], or sperm component factors [48]. As the father’s environment modification happened during the father’s embryonic development in our study, we hypothesize that epigenetic effects may be involved in the results observed here.

## Conclusions

Our results indicate that the females’ diets can affect their grand-offspring through their sons, in the Muscovy duck. This was observed in two genetic backgrounds (hybrids and purebreds) for traits such as body weight before force-feeding and triglycerides plasma concentration after force-feeding, whereas other traits affected by the grand-mother’s diet were genetic background-specific, such as the weight gain during force-feeding or the localization of lipid deposition. Whether this transmission through the father of information induced in the grand-mother by the environment is epigenetic remains to be demonstrated.

## Availability of supporting data

The data sets supporting the results of this article are included as Additional file 2.

## Additional files

Additional file 1:
**Results obtained on G2 offspring by genetic type, sex and grand-maternal diet.** (DOCX 41 kb) Additional file 2:
**Supporting data.** (TXT 27 kb)

## Abbreviations

FF: Force feeding
GMMD: Grand-maternal met-deficiency
VLDL: Very low density lipoproteins
