# Supplementary material for: Influence of grand-mother diet on offspring performances through the male line in Muscovy duck
Source: BMC Genet. 2015 Dec 21;16:145. doi: 10.1186/s12863-015-0303-z (PMC4687110; doi:10.1186/s12863-015-0303-z)
Supplement: Additional file 1: — Results obtained on G2 offspring by genetic type, sex and grand-maternal diet. (DOCX 41 kb) [file 12863_2015_303_MOESM1_ESM.docx]

**Additional Table1. Results obtained on G2 offspring by genetic type, sex and grand-maternal diet**

|  | Mule ducks | | | | Muscovy ducks | | | |
| --- | --- | --- | --- | --- | --- | --- | --- | --- |
|  | Males | | Females | | Males | | Females | |
|  | Met-deficient | Control | Met-deficient | Control | Met-deficient | Control | Met-deficient | Control |
| Growth traits  Body weight, 4 wks  Body weight, 8 wks  Body weight, 12 wks  Weight gain, 4-8wks  Weight gain, 8-12wks  Weight gain, 4-12wks  Force-feeding traits FF  Weight gain (WG) in FF  Body weight end FF  Feed consumption (FC)  Ratio (FC/WG) | 1421±14  2924±26  3433±31  1502±21  509±50  2012±29  1678±32  5130±42  14188±66  8.48±0.21 | 1498±15  3003±28  3463±34  1504±23  460±28  1957±32  1581±33  5045±45  14169±71  9.04±0.22 | 1231±13  2704±25  3347±33  1473±13  546±26  2115±29  1541±31  4903±41  13954±64  9.08±0.20 | 1294±17  *28*27±32  3458±25  1532±25  632±32  2164±36  1422±38  4880±50  13880±79  10.13±0.25 | 1660±28  4347±51  5269±59  2678±37  921±50  3609±55  1370±42  6640±71  -  - | 1704±28  4300±48  5501±58  2596±36  1202±49  3798±54  1379±43  6899±71  -  - | 1183±29  2715±52  2955±61  1525±38  227±51  1772±57  893±43  3849±73  -  - | 1232±28  *28*22±49  2941±59  1589±37  119±50  1708±55  926±43  3881±73  -  - |

**Additional Table1 (suite). Results obtained on G2 offspring by genetic type, sex and grand-maternal diet**

|  | Mule ducks | | | | Muscovy ducks | | | |
| --- | --- | --- | --- | --- | --- | --- | --- | --- |
|  | Males | | Females | | Males | | Females | |
|  | Met-deficient | Control | Met-deficient | Control | Met-deficient | Control | Met-deficient | Control |
| Carcass traits  Carcass weight (CW)  Magret weight  Ratio (magret/carcass)  Magret muscle weight  Ratio (fat skin/magret)  Abd. fat weight (AF)  Ratio AF/CW (%)  Liver weight (LW)  Ratio (LW/CW)  Liver melting rate  Blood metabolites  Glucose mid-FF (g/L)  Glucose end-FF (g/L)  Trigly. mid-FF (g/L)  Trigly. end-FF (g/L) | 4614±40  372.2±4.7  8.06±0.09  234.6±3.1  36.9±0.5  163.6±3.7  3.53±0.08  535±18  11.6±0.4  10.5±0.9  1.95±0.09  2.40±0.25  0.42±0.02  0.77±0.08 | 4539±42  370.3±4.9  8.12±0.09  232.6±3.2  37.0±0.6  149.5±3.8  3.28±0.08  526±19  11.6±0.4  10.8±0.9  1.94±0.10  2.30±0.24  0.49±0.02  0.57±0.08 | 4441±38  361.9±4.5  8.15±0.08  226.2±2.9  37.5±0.5  149.8±3.5  3.37±0.07  560±17  12.6±0.4  13.1±0.8  2.22±0.09  4.15±0.25  0.46±0.02  0.93±0.09 | 4411±47  358.6±5.6  8.14±0.10  227.5±3.7  36.5±0.6  144.0±4.3  3.27±0.09  544±22  12.3±0.4  14.0±1.0  2.23±0.11  3.24±0.32  0.42±0.02  0.72±0.11 | 6073±63  563±7  9.27±0.11  401±6  28.8±0.7  184.0±7.6  3.04±0.14  557±16  9.2±0.3  -  2.21±0.15  2.49±0.19  0.32±0.02  0.46±0.08 | 6270±62  582±7  9.27±0.10  416±6  28.6±0.5  192.4±7.6  3.10±0.14  517±16  8.3±0.3  -  2.10±0.14  2.20±0.19  0.31±0.02  0.38±0.08 | 3517±65  311±8  8.86±0.11  223±7  28.2±0.7  124.5±7.9  3.53±0.15  402±17  11.4±0.3  -  2.47±0.15  2.68±0.20  0.50±0.02  0.71±0.08 | 3523±65  322±8  9.14±0.11  227±7  29.3±0.7  117.0±7.9  3.30±0.15  384±17  10.9±0.3  -  2.22±0.15  2.82±0.20  0.35±0.02  0.42±0.08 |
